# Distal Radial Artery Access for Recanalization of Radial Artery Occlusion and Repeat Intervention: A Single Center Experience

**DOI:** 10.3390/jcm11236916

**Published:** 2022-11-23

**Authors:** Alexandru Achim, Kornél Kákonyi, Zoltán Jambrik, Dorottya Olajos, Attila Nemes, Olivier F. Bertrand, Zoltán Ruzsa

**Affiliations:** 1 Internal Medicine Department, Division of Invasive Cardiology, University of Szeged, 6720 Szeged, Hungary; 2 Bács-Kiskun County Hospital, Teaching Hospital of the Szent-Györgyi Albert Medical University, 6725 Kecskemét, Hungary; 3 Cardio-Thoracic Center, University Laval, Quebec City, QC G1V 0A6, Canada

**Keywords:** radial artery occlusion, radial recanalization, distal radial access

## Abstract

**Background**: Once occluded, the radial artery becomes unsuitable for repeat interventions and obligates the need for alternative vascular access, such as the femoral approach, which is not encouraged by current guidelines. With the dissemination of distal radial access (DRA), which allows the cannulation of the artery in its distal segment and which remains patent even in the case of radial artery occlusion (RAO), the option to perform angioplasty at this level becomes feasible. **Methods**: Thirty patients with RAO were enrolled in this pilot study. Recanalization was performed through DRA using hydrophilic guidewires. The feasibility endpoint was procedural success, namely the successful RAO recanalization, the efficacy endpoint was patency of the artery at 30 days, and the safety endpoint was the absence of periprocedural vascular major complications or major adverse cardiac and cerebrovascular events. **Results**: The mean age of the patients was 63 ± 11 years, and 15 patients (50%) were men. Most patients had asymptomatic RAO (*n* = 28, 93.3%), and only two (6.6%) reported numbness in their hands. The most common indication for the procedure was PCI (19, 63.2%). Total procedural time was 41 ± 22 min, while the amount of contrast used was 140 ± 28 mL. Procedural success was 100% (*n* = 30). Moreover, there were no major vascular complications (0%); only two small hematomas were described (10%) and one had an angiographically visible perforation (3%). One case of periprocedural stroke was reported (3%), with onset immediately after the procedure and recovering 24 h later. Twenty-seven radial arteries (90%) remained patent at the one-month follow-up. **Conclusions**: RAO recanalization is feasible and safe, and by using dedicated hydrophilic guidewires, the success rate is high without significantly increasing procedural time or the amount of used contrast.

## 1. Introduction

The distal radial access (DRA), a vascular access which now is established and widely adopted in many types of transcatheter interventions [1,2,3,4], was firstly described as a way of accessing and recanalizing the occluded proximal radial artery segment [5]. Because of the superficial palmar arch, which takes off proximal to the emergence of the distal RA, this segment remains patent by collateralization, making puncture and retrograde radial angioplasty feasible [6].

Radial artery occlusion (RAO) represents one of the few remaining limitations of radial arterial access for coronary procedures. Late radial artery thrombosis occurs in 3–9% of cases after transradial interventions [7], and chronic RAO (>30 days) is discouraged for future attempts at vascular access at this site [8]. Once occluded, the artery becomes unsuitable for repeat interventions and leads to the need for alternative vascular access, such as the left radial artery (with some risk of bilateral RAO) or, contrary to current guidelines, the femoral artery with its shortcomings (prolonged hemostasis, immobilization, etc.) and complications (large groin hematoma, arterio-venous fistula or false aneurysm, retroperitoneal hemorrhage, etc.) [9]. By 2021, both U.S. and European guidelines have converged to advocate radial access for all coronary interventions, whether stable or acute [10,11]. Therefore, there is a contemporary focus on performing these interventions via the radial artery, although it must be recognized that the safety of the femoral approach has also improved by the large-scale introduction of ultrasound-guided puncture and vascular closure devices. Nevertheless, reopening a vascular access considered abolished represents an advantage for the operator.

With the adoption of DRA, attempts to recanalize recent or chronic RAO have also increased, with promising results, but reporting is limited to case reports or case series [12,13,14,15]. The conclusion of these publications called for larger registries to investigate the prevalence of complications of radial angioplasty (perforation, hematoma, digital ischemia, etc.) [12] and its long-term patency [13,14], which is perhaps the most important aspect of the prospect of reintervention, especially since RAO is asymptomatic in most cases and recanalization performed exclusively for hand ischemia is less common [15]. The aim of this study was to explore the feasibility, safety, and long-term patency of the recanalization of chronic RAO and to present some technical procedural insights.

## 2. Methods

### 2.1. Study Population

The present study was a proof-of-concept study. For pilot studies, at least 30 patients are recommended [16]. Thus, from July 2016 to March 2022, we prospectively enrolled 30 consecutive patients with RAO, the majority who presented in our catheterization laboratory for suspected or known coronary artery disease and who received DRA. The cohort was recruited regardless of comorbidities or the severity of coronary heart disease. Of course, cases of hemodynamic instability, such as STEMI/NSTEMI or cardiogenic shock as well as cases of hypoplastic radial artery (seen on ultrasound or known from documents), were excluded due to time-to-revascularization reasons. The decision not to change the access site and to proceed with radial angioplasty was made by the operator in charge; all procedures were performed by two operators experienced in DRA. An ad hoc informed consent for the procedure was obtained from all patients. The study protocol was approved by the Ethics Committee of the Affiliated Second Internal Medicine Department Hospital of Szeged University.

### 2.2. Procedural Protocol and Technical Pearls

The right or left upper arm was positioned in a semi-pronated manner. Because our center’s protocol requires ultrasound-guided DRA for all patients since 2019, the diagnosis of RAO was made even during the preparation for puncture. Antegrade damped Doppler sign or reverse flow through the palmar arch could be seen directly.

**Figure 1:**
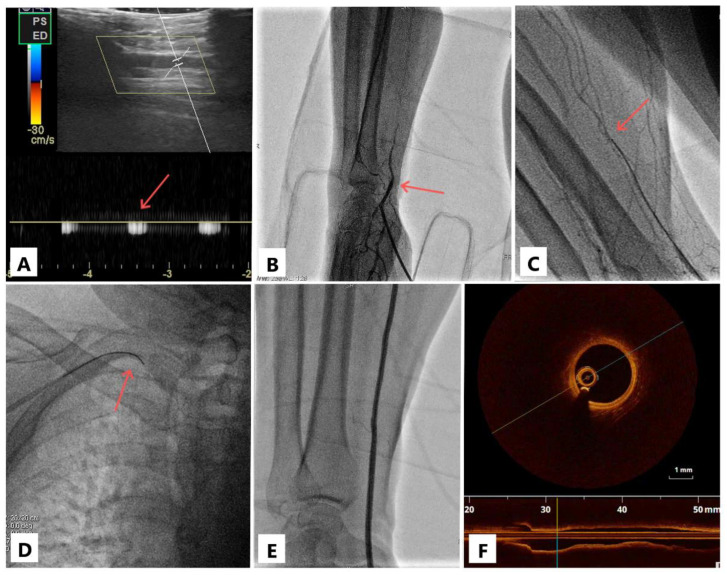
Case illustration. Doppler sign in the distal radial artery (panel (**A**), arrow); visualization of the radial artery occlusion (panel (**B**), arrow); the occlusion passage with a 0.018″ guidewire (panel (**C**), arrow) on which the sheathless catheter is then advanced (panel (**D**), arrow); after coronary angioplasty, the radial artery is visualized both with contrast (panel (**E**)) and by optical coherence tomography (panel (**F**)); note the three-layered structure of the arterial wall (intima, media, adventitia) suggestive of a normal morphology.

The details of DRA puncture are described elsewhere [3], but briefly, on a 21-gauge needle, the intraluminal access was confirmed by the ultrasound probe and not by the palpation of the pulse. Directly through the needle, the contrast was injected to visualize the occlusion (Figure 1). If partial advancement of the standard puncture guidewire (0.018″) was possible (for more proximal occlusions), the 5- or 6-French sheath could be partially inserted, and angiography was performed through it. On a case-by-case basis, the wiring was then continued either via the 21-gauge needle or via the introducer needle inserted into the sheath valve. Under fluoroscopy, dedicated polymer-jacket hydrophilic chronic total occlusion (CTO) coronary or peripheral guidewires of 0.014″ or 0.018″ (Gladius 0.014, Gladius 0.018, [Asahi Intecc, Nagoya, Japan] Pilot 50 0.014 or Progress 40 0.014 [Abbott Vascular Inc., Temecula, CA, USA]) were advanced through the occlusion, either by intraluminal tracking (true lumen, through CTO microchannels) or by subintimal tracking and reentry, which can be easily performed with the Gladius knuckle.

Of the two options above, the most favorable was chosen on an ad hoc basis depending on the level of occlusion and the possibility to advance the guidewire because the sheath provides support. In our experience, the safest wiring option would be directly through the needle with hydrophilic guidewires with a heavier tip-load—a good and fast success rate has been observed with these guidewires. Some puncture needles come with a plastic cannula that can be secured to the skin with sterile tape. Subsequently, a Y-connector can be attached directly to the cannula and the guidewire advanced through the respective tube.

When the tip of the wire freely entered the brachial artery, a sheathless catheter was advanced over the wire through the occlusion by a push-and-rotate maneuver (similar to “dotterization”). If we had encountered resistance, we would have added the sheath for support, but sheathless catheters were always preferred. The 0.018″ wire provides more support, and the balloon-assisted tracking technique may be useful when negotiating the sheathless catheter over the 0.014″ guidewire which offers less support. Subsequently, the coronary procedure was performed. Along with the learning curve, it was found that advancement of the sheathless system was sufficient to achieve RAO recanalization and eliminate the need for balloon dilatation. Finally, patent hemostasis was obtained; no doses other than standard periprocedural heparin were administered.

### 2.3. Follow-Up and Endpoint

The **feasibility endpoint** was a procedural success, namely successful flow restoration from the brachial artery all along the radial artery until the two palmar arches. Flow restoration was quantified by angiography at the end of the procedure and only stable, normal flow (the equivalent of TIMI III) was considered a procedural success. In some cases, this qualitative evaluation was difficult to put in the context in which the sheath occludes the vessel at the point of insertion; nevertheless, the fact that the radial artery was patent up to its level with good backflow through the sheath was considered a procedural success as well, which was also confirmed by duplex ultrasound at follow-up. The **efficacy endpoint** was patency of the artery at 30 days, verified by ultrasound Doppler signal. Pulse palpation was considered unreliable as through abundant collaterals formation; it is possible to have complete occlusion of the radial artery in the presence of an easily palpable radial pulse at the wrist. The **safety endpoint** was a composite of the absence of (i) intra- or periprocedural major complications (hematoma EASY 3–4 [17], digital ischemia, thrombus migration to brachial/ulnar artery, and compartment syndrome) and (ii) major adverse cardiac and cerebrovascular events (MACCE) defined as transient ischemic attack, stroke, myocardial infarction, and cardiac-related death. These outcomes were evaluated up to the moment of discharge.

### 2.4. Statistical Analysis

Continuous data were tested for normal distribution with the Kolmogorov–Smirnov test. Normally distributed variables are presented as mean ± standard deviation and were compared by *t*-test. Otherwise, they are presented as median and interquartile range, and the Mann–Whitney U test was used. Categorical variables are summarized in terms of numbers, and percentages and were compared by using the chi-squares test. Statistical analysis was performed using the statistical software program SPSS (version 22.0; SPSS Inc., Chicago, IL, USA). A probability value of <0.05 was considered statistically significant.

## 3. Results

Thirty consecutive patients with RAO were enrolled in our study. All were punctured by DRA. The demographic data are presented in Table 1. The mean age of the patients was 63 ± 11 years, and 15 patients (50%) were men. Almost the entire cohort was known to have coronary artery disease (*n* = 27, 90%). Among the study population, 21 patients (70%) received right DRA, and the most common indication for the procedure was percutaneous coronary intervention (PCI) (19, 63.2%). Most patients had asymptomatic RAO (*n* = 28, 93.3%); only two (6.6%) reported numbness in their hand.

The procedural characteristics and success rate of DRA RAO recanalization are listed in Table 2. Procedural success through retrograde wiring was 100% (*n* = 30), and all cases (*n* = 30, 100%) were performed by the “dottering” technique with the sheathless system. Moreover, there were no major vascular complications (0%); only two EASY 1–2 hematomas were described (10%), and one had an angiographically visible perforation (3%). The remaining dissections were several (*n* = 16, 53%) but were not flow-limiting. All patients had a good flow at the end of the procedure when a final upper limb angiography was performed by continuous injection while withdrawing the catheter. One case of periprocedural stroke was reported (3%) with onset immediately after the procedure (after sheath removal) and recovering 24 h later. As for efficacy endpoint, 27 of the radial arteries (90%) remained patent at the one-month follow-up.

All occlusions were chronic (*n* = 30, 100%), most being recanalized using 0.018″ hydrophilic guidewires (53%). Approximately one-quarter of recanalization cases (23%) required the use of more than one type of guidewire, and the most commonly used guidewire was Gladius (Asahi Intecc, Nagoya, Japan) (either 0.018″ or 0.014″) in 63% of cases. None of the cases required the administration of a local thrombolytic agent or mechanical thrombaspiration. The mean amount of contrast material used was 140 ± 28 mL, and the mean procedure time was 41 ± 22 min; these measurements incorporated the entire intervention.

## 4. Discussion

**Figure 2:**
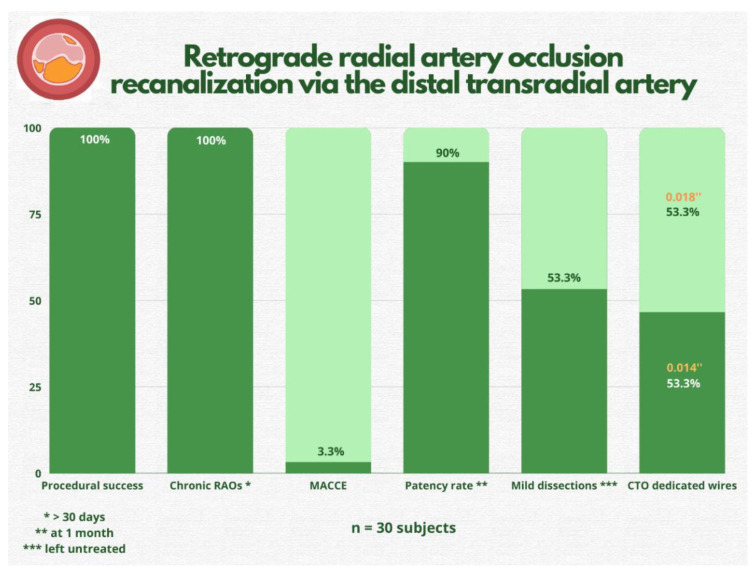
Study’s most important findings. RAO = radial artery occlusion; MACCE = major adverse cardiac and cerebrovascular events; CTO = chronic total occlusion.

The main findings of our study were that **(1)** RAO recanalization by DRA is safe and has a high success rate, **(2)** radial angioplasty by subintimal tracking and reentry is straightforward and does not require a large amount of time or contrast, **(3)** long-term patency remains high (90% at one-month follow-up), and **(4)** the rate of associated vascular complications is low (Figure 2). The only major event was a stroke after the procedure, which, however, cannot be irrefutably attributed to radial artery recanalization (migration of thrombotic material through the catheter, etc.). The stroke occurred immediately after completion of PCI; thus, it can be labeled as periprocedural, and the mechanism of embolization remains unknown. Urgent computed tomography angiography showed no large vessel occlusion (internal carotid artery and middle cerebral artery), and after 24 h, the patient had fully recovered from symptoms.

To our knowledge, only four previous reports tested the feasibility of RAO recanalization via DRA [12,13,14,15]. Gasparini et al. described the same technique of balloonless dilatation and wiring directly through the needle [12]. Of the seven patients, five were successfully recanalized (71% success rate), choosing solely 0.014″ polymer-jacket coronary guidewires. The subintimal tracking and reentry technique was used in 80% of their cases [12]. On the other hand, Ali et al.’s patient complained of hand claudication after the first intervention, initially RAO being treated with an anticoagulant for four weeks without improvement [15]. This shows that sometimes the indication for RAO angioplasty is dictated by the patient’s symptoms [18]. Subsequently, the occlusion was treated by balloon dilation, but a large amount of floating thrombus was eventually treated with a tissue plasminogen activator and repeated passages of thrombaspiration. Moreover, the patient was discharged with six months of planned apixaban therapy. In such cases of relatively recent thrombosis, a tailored drug-interventional treatment is therefore useful. Moreover, the techniques were embraced by angiologists as well. In a recent report of a case of acute embolic occlusion of both radial and ulnar arteries leading to acute upper limb ischemia, DRA proved to be the last resort in accessing and opening the vessel retrogradely [19].

Two meta-analyses showed similar rates of successful cannulation between DRA and conventional transradial approach [20,21]. Moreover, no major safety issue has been reported so far among published studies. They pointed out a very low incidence of radial artery spasm, shorter time to hemostasis, and a substantial absence of forearm RAO with DRA despite no mention of any dedicated strategy to favor vessel patency [22]. Ultrasound guidance ensures successful distal radial puncture and lower complications. In the only study comparing ultrasound-guided to conventional DRA, ultrasound guidance increased the success of DRA from 87% to 97% [23]. However, there are other positive signals, e.g., in a recent study demonstrating no hand function impairment by DRA, ultrasound guidance was used in the majority of cases (80%) [24].

Recently, several methods to minimize the risk of RAO have been reviewed in an international consensus [25]. This document supports systematic implementation of these methods in the everyday interventional practice. One of them was DRA, which has been proposed as a potential approach to prevent RAO because of its anatomical basis and physiological rationale [25]. This fact becomes even more relevant after the publication of two recent studies that showed RAO is not affected by the low dose of rivaroxaban (10 mg) administered shortly after the procedure or by the systematic administration of nitroglycerin at the beginning and end of the procedure [26,27]. Of note, in an experimental study in healthy volunteers, simulated occlusion of the distal radial artery in the anatomic snuffbox did not cause significant flow reduction in the forearm radial artery compared with simulated occlusion of the radial artery at the wrist [28]. This detail explains the asymptomatic occlusion and underscores the importance of preserving the artery as a medical investment rather than having an immediate clinical impact. With the recent results of the DISCO trial, the belief that DRA may lower RAO rates is still debatable but certainly DRA has a role in re-accessing the artery in cases of RAO [29].

A significant percentage of our cases (30%) had calcific deposits in the radial arterial wall; it is plausible that the poor condition of the artery contributes to post-PCI occlusion. Not surprisingly, radial calcification has been correlated with coronary calcification, reflecting the systemic nature of the atherosclerotic disease [30]. However, in the case of intravascular optical coherence tomography imaging (Figure 1F), the artery appears healthy. This shows that thrombosis can also occur in perfectly normal radial arteries if the hemostasis is not achieved correctly or the number of puncture attempts is high. It has been observed that repeated traumatic penetration of the arterial wall leads to micro-hematomas in the arterial wall, which can stretch intraluminal and occlude the vessel (extrinsic compression) [3].

Our findings are clinically important for several reasons. First, it provides evidence and confidence that the same coronary CTO skills and techniques can be applied to any occluded artery, and in fact, the radial artery CTO is not a futile attempt, and DRA represents a big advantage in this regard. Second, there is no need for balloon angioplasty to dilate the radial artery occlusion, as inserting a sheathless catheter over the 0.0018″ CTO guidewire appears capable of achieving radial recanalization. When “dottering” the radial artery with sheathless catheters, we found no risk of injury to the vessel wall, and advancement of the system should not require a significant force. Balloon dilatation offers the same result, but with more steps to perform along the way (repeated inflations, more radiation, contrast injections) that the operator could avoid. In a randomized comparison between a 6.5-French sheathless catether versus a conventional 6-French sheath and catheters, Horie et al. found no difference in major adverse events with a lower incidence of RAO and arterial spasm with sheathless catheters [31]. Third, once recanalized, the artery’s long-term patency rate is high (90%). Nevertheless, the artery should not be used by surgeons as bypass conduit for coronary revascularization or as radial artery–cephalic vein arteriovenous fistula for patients on chronic hemodialysis, both being risky options without any supporting evidence [32,33].

We acknowledge that the study has one significant limitation that it is being conducted in one center with all procedures being performed by two operators. Hence, its large-scale application may be limited. Nevertheless, the techniques are encouraging.

In summary, transcatheter interventions are becoming increasingly complex, and the chronic nature of coronary artery disease forces patients to undergo repeated interventions throughout their lives. In this context, vascular access becomes as important as the intervention itself, and special attention must be paid to this part of the procedure and its education among the operators. Of course, RAO prevention remains desirable. Patent hemostasis should be universally indicated, and ultimately, radial artery recanalization via DRA may reopen “closed doors” for selected patients.

## 5. Conclusions

RAO recanalization is feasible and safe, and by using dedicated CTO 0.014″ or 0.018″ guidewires, the success rate is high without significantly increasing the procedure time or the amount of contrast agent used. The rationale for this procedure has its place in the momentum of expanding indications and skillsets for percutaneous procedures and emphasizing their performance via the transradial approach.

## Figures and Tables

**Table 1:** Demographic and clinical data.

		*n* (%)
**Demographic data**	Age (years)	63.2 ± 11.2
	Male	15 (50)
	Weight (kg)	78.7 ± 15.7
	Height (cm)	159.1 ± 8.9
	Smoking	7 (23.3)
	Hypertension	18 (60)
	Renal insufficiency	7 (23.3)
	Dyslipidemia	14 (46.6)
	Atrial fibrillation	9 (30)
	COPD	4 (13.3)
	Diabetes mellitus	12 (40)
	CAD	27 (90)
	PAD	14 (46.6)
**Procedure**	Coronary angiography	9 (30)
	PCI	17 (56.6)
	CTO PCI	2 (6.6)
	TAVI (secondary access)	1 (3.3)
	Carotid stenting	1 (3.3)
**Vascular access**	Right distal radial artery	21 (70)
	Left distal radial artery	9 (30)

COPD = chronic obstructive pulmonary disease; CAD = coronary artery disease; PAD = peripheral artery disease; PCI = percutaneous coronary intervention; CTO = chronic total occlusion; TAVI = transcatheter aortic valve implantation.

**Table 2:** Procedural results and outcomes. Vascular major complication was defined as hematoma EASY 3–4, digital ischemia, thrombus migration to brachial/ulnar artery, and compartment syndrome.

	Pre-Interventional *n* (%)	Post-Interventional *n* (%)	
**Symptoms**			*p*-*value*
**Pain**	0	0	*1.0*
**Numbness**	2 (6.6)	0	*0.12*
**Weakness**	0	0	*1.0*
**Asymptomatic RAO**	28 (93.3)	28 (93.3)	*1.0*
**Vascular ultrasound (radial site)**			
Radial artery (mm)	2.1 ± 0.5	2.1 ± 0.5	*1.0*
Hematoma (EASY 1–2)	0	3 (10)	*0.92*
Hematoma (EASY 3–4)	0	0	*1.0*
Radial artery occlusion	30 (100)	0	*0.001*
Pseudoaneurysm	0	0	*1.0*
**Wire used**			
0.014″ hydrophilic		14 (46.6)	
0.018″ hydrophilic		16 (53.3)	
**PTA result**			
Good final flow		30 (100)	
Dissection		16 (53.3)	
Perforation		1 (3.3)	
Thrombus migration		0 (0)	
**Occlusion**			
Length (mm)	50 ± 60		
Calcific vessel	9 (30)		
CTO	30 (100)		
**Endpoints**			
Procedural success		30 (100)	
Vascular major complications *		0 (0)	
MACCE		1 (3.3)	
1-month follow-up			
Re-occlusion		3 (10)	

RAO = radial artery occlusion; PTA = percutaneous transluminal angioplasty; CTO = chronic total occlusion; MACCE = major adverse cardiac and cerebrovascular events; * vascular major complications were defined as: EASY 3–4 hematoma, digital ischemia, thrombus migration to brachial/ulnar artery, and compartment syndrome.

## Data Availability

The datasets generated during and/or analysed during the current study are available from the corresponding author on reasonable request.

## Abbreviations

DRA	distal radial access
RAO	radial artery occlusion
CTO	chronic total occlusion
MACCE	major adverse cardiac and cerebrovascular events
PCI	percutaneous coronary intervention

## References

[1] Achim A., Szűcsborus T., Sasi V., Nagy F., Jambrik Z., Nemes A., Varga A., Homorodean C., Bertrand O.F., Ruzsa Z. (2022). Safety and Feasibility of Distal Radial Balloon Aortic Valvuloplasty: The DR-BAV Study. JACC Cardiovasc. Interv..

[2] Achim A., Szűcsborus T., Sasi V., Nagy F., Jambrik Z., Nemes A., Varga A., Bertrand O.F., Ruzsa Z. (2022). Distal Radial Secondary Access for Transcatheter Aortic Valve Implantation: The Minimalistic Approach. Cardiovasc. Revasc. Med..

[3] Achim A., Kákonyi K., Jambrik Z., Nagy F., Tóth J., Sasi V., Hausinger P., Nemes A., Varga A., Bertrand O.F. (2021). Distal Radial Artery Access for Coronary and Peripheral Procedures: A Multicenter Experience. J. Clin. Med..

[4] Achim A., Szigethy T., Olajos D., Molnár L., Papp R., Bárczi G., Kákonyi K., Édes I.F., Becker D., Merkely B. (2022). Switching from Proximal to Distal Radial Artery Access for Coronary Chronic Total Occlusion Recanalization. Front. Cardiovasc. Med..

[5] Babunashvili A., Dundua D. (2011). Recanalization and reuse of early occluded radial artery within 6 days after previous transradial diagnostic procedure. Catheter. Cardiovasc. Interv..

[6] Sgueglia G.A., Di Giorgio A., Gaspardone A., Babunashvili A. (2018). Anatomic Basis and Physiological Rationale of Distal Radial Artery Access for Percutaneous Coronary and Endovascular Procedures. JACC Cardiovasc. Interv..

[7] Stella P.R., Kiemeneij F., Laarman G.J., Odekerken D., Slagboom T., van der Wieken R. (1997). Incidence and outcome of radial artery occlusion following transradial artery coronary angioplasty. Catheter. Cardiovasc. Diagn..

[8] Kotowycz M.A., Dzavík V. (2012). Radial artery patency after transradial catheterization. Circ. Cardiovasc. Interv..

[9] Shroff A.R., Gulati R., Drachman D.E., Feldman D.N., Gilchrist I.C., Kaul P., Lata K., Pancholy S.B., Panetta C.J., Seto A.H. (2020). SCAI expert consensus statement update on best practices for transradial angiography and intervention. Catheter. Cardiovasc. Interv..

[10] Lawton J.S., Tamis-Holland J.E., Bangalore S., Bates E.R., Beckie T.M., Bischoff J.M., Bittl J.A., Cohen M.G., DiMaio J.M., Don C.W. (2022). 2021 ACC/AHA/SCAI Guideline for Coronary Artery Revascularization: A Report of the American College of Cardiology/American Heart Association Joint Committee on Clinical Practice Guidelines. Circulation.

[11] Neumann F.J., Sousa-Uva M., Ahlsson A., Alfonso F., Banning A.P., Benedetto U., Byrne R.A., Collet J.P., Falk V., Head S.J. (2019). 2018 ESC/EACTS Guidelines on myocardial revascularization. Eur. Heart J..

[12] Sanz-Sánchez J., Regazzoli D., Petriello G., Leone P.P., Reimers B., Gasparini G.L. (2021). The Last Broken Barrier: Retrograde Radial Artery Recanalization Prior to Transradial Coronary Interventions. Cardiovasc. Revasc. Med..

[13] Li F., Shi G.W., Zhang B.F., Yu X.L., Huang H.M., Xiao J.Q., Cai G.J. (2021). Recanalization of the occluded radial artery via distal transradial access in the anatomic snuffbox. BMC Cardiovasc. Disord..

[14] Alkhawam H., Windish S., Abo-Salem E. (2019). Distal radial artery access among cases with radial artery occlusion for primary percutaneous intervention. Future Cardiol..

[15] Ali S., Abdullah M.S., Abdelrahman K., Ali A., Faisal F., Ali A. (2020). Total Radial Artery Occlusion Following Transradial Access: Complete Recanalization via the Anatomical Snuffbox. Methodist Debakey Cardiovasc. J..

[16] Lancaster G.A., Dodd S., Williamson P.R. (2004). Design and analysis of pilot studies: Recommendations for good practice. J. Eval. Clin. Pract..

[17] Bertrand O.F. (2010). Acute forearm muscle swelling post transradial catheterization and compartment syndrome: Prevention is better than treatment. Catheter. Cardiovasc. Interv..

[18] Ruzsa Z., Berta B., Tóth J., Nemes B., Katona A., Hüttl A., Ungi I., Bertrand O.F., Merkely B. (2019). Short- and long-term results with a percutaneous treatment in critical hand ischaemia. Catheter. Cardiovasc. Interv..

[19] Giusca S., Schmidt A., Korosoglou G. (2022). A case report of distal radial puncture in a patient with acute upper limb ischaemia: The last hope of the cardiologist?. Eur. Heart J. Case Rep..

[20] Prasad R.M., Pandrangi P., Pandrangi G., Yoo H., Salazar A.M., Ukponmwan E., Kehdi M., Abela G. (2022). Meta-Analysis Comparing Distal Radial Artery Approach Versus Traditional for Coronary Procedures. Am. J. Cardiol..

[21] Cao J., Cai H., Liu W., Zhu H., Cao G. (2021). Safety and Effectiveness of Coronary Angiography or Intervention through the Distal Radial Access: A Meta-Analysis. J. Interv. Cardiol..

[22] Sgueglia G.A., Lee B.K., Cho B.R., Babunashvili A., Lee J.B., Lee J.W., Schenke K., Lee S.Y., Harb S. (2021). Distal Radial Access: Consensus Report of the First Korea-Europe Transradial Intervention Meeting. JACC Cardiovasc. Interv..

[23] Mori S., Hirano K., Yamawaki M., Kobayashi N., Sakamoto Y., Tsutsumi M., Honda Y., Makino K., Shirai S., Ito Y. (2020). A Comparative Analysis between Ultrasound-Guided and Conventional Distal Transradial Access for Coronary Angiography and Intervention. J. Interv. Cardiol..

[24] Sgueglia G.A., Hassan A., Harb S., Ford T.J., Koliastasis L., Milkas A., Zappi D.M., Navarro Lecaro A., Ionescu E., Rankin S. (2022). International Hand Function Study Following Distal Radial Access: The RATATOUILLE Study. JACC Cardiovasc. Interv..

[25] Bernat I., Aminian A., Pancholy S., Mamas M., Gaudino M., Nolan J., Gilchrist I.C., Saito S., Hahalis G.N., Ziakas A. (2019). Best Practices for the Prevention of Radial Artery Occlusion After Transradial Diagnostic Angiography and Intervention: An International Consensus Paper. JACC Cardiovasc. Interv..

[26] Liang D., Lin Q., Zhu Q., Zhou X., Fang Y., Wang L., Xiang G., Zheng K.I., Huang W., Shan P. (2022). Short-Term Postoperative Use of Rivaroxaban to Prevent Radial Artery Occlusion after Transradial Coronary Procedure: The RESTORE Randomized Trial. Circ. Cardiovasc. Interv..

[27] da Silva R.L., de Andrade P.B., Dangas G., Joaquim R.M., da Silva T.R.W., Vieira R.G., Pereira V.C., Sousa A.G.M., Feres F., Costa J.R. (2022). Randomized Clinical Trial on Prevention of Radial Occlusion After Transradial Access Using Nitroglycerin: PATENS Trial. JACC Cardiovasc. Interv..

[28] Sgueglia G.A., Santoliquido A., Gaspardone A., Di Giorgio A. (2021). First Results of the Distal Radial Access Doppler Study. JACC Cardiovasc. Imaging..

[29] Aminian A., Sgueglia G.A., Wiemer M., Kefer J., Gasparini G.L., Ruzsa Z., van Leeuwen M.A.H., Ungureanu C., Leibundgut G., Vandeloo B. (2022). Distal Versus Conventional Radial Access for Coronary Angiography and Intervention: The DISCO RADIAL Trial. JACC Cardiovasc. Interv..

[30] Achim A., Kákonyi K., Nagy F., Jambrik Z., Varga A., Nemes A., Chan J.S.K., Toth G.G., Ruzsa Z. (2022). Radial Artery Calcification in Predicting Coronary Calcification and Atherosclerosis Burden. Cardiol. Res. Pract..

[31] Horie K., Tada N., Isawa T., Matsumoto T., Taguri M., Kato S., Honda T., Ootomo T., Inoue N. (2018). A randomised comparison of incidence of radial artery occlusion and symptomatic radial artery spasm associated with elective transradial coronary intervention using 6.5 Fr SheathLess Eaucath Guiding Catheter vs. 6.0 Fr Glidesheath Slender. Eurointervention.

[32] Achim A., Leibundgut G. (2022). FAME 3 fails to defame coronary artery bypass grafting: What went wrong in the percutaneous coronary intervention arm?. Eur. J. Cardiothorac. Surg..

[33] Nappi F., Bellomo F., Nappi P., Chello C., Iervolino A., Chello M., Acar C. (2021). The Use of Radial Artery for CABG: An Update. Biomed Res. Int..

